# The Effective Combination between 3D Cancer Models and Stimuli-Responsive Nanoscale Drug Delivery Systems

**DOI:** 10.3390/cells10123295

**Published:** 2021-11-25

**Authors:** Federica Foglietta, Loredana Serpe, Roberto Canaparo

**Affiliations:** Department of Drug Science and Technology, University of Torino, Via Pietro Giuria 13, 10125 Torino, Italy; federica.foglietta@unito.it (F.F.); roberto.canaparo@unito.it (R.C.)

**Keywords:** drug delivery systems (DDSs), three-dimensional (3D) cancer models, endogenous stimuli-responsive DDSs, exogenous stimuli-responsive DDSs

## Abstract

Stimuli-responsive drug-delivery systems (DDSs) have emerged as a potential tool for applications in healthcare, mainly in the treatment of cancer where versatile nanocarriers are co-triggered by endogenous and exogenous stimuli. Two-dimensional (2D) cell cultures are the most important in vitro model used to evaluate the anticancer activity of these stimuli-responsive DDSs due to their easy manipulation and versatility. However, some limitations suggest that these in vitro models poorly predict the outcome of in vivo studies. One of the main drawbacks of 2D cell cultures is their inadequate representation of the 3D environment’s physiological complexity, which sees cells interact with each other and the extracellular matrix (ECM) according to their specific cellular organization. In this regard, 3D cancer models are a promising approach that can overcome the main shortcomings of 2D cancer cell cultures, as these in vitro models possess many peculiarities by which they mimic in vivo tumors, including physiologically relevant cell–cell and cell–ECM interactions. This is, in our opinion, even more relevant when a stimuli-responsive DDS is being investigated. In this review, we therefore report and discuss endogenous and exogenous stimuli-responsive DDSs whose effectiveness has been tested using 3D cancer cell cultures.

## 1. Introduction

Three-dimensional (3D) cell cultures have acquired considerable amounts of interest over the last twenty years becoming a versatile tool, especially for cancer research, due to their intrinsic capacity to better mimic the tumor microenvironment (TME) complexity [1,2]. Currently, the growth of 3D cell cultures can be generated using several protocols, which allows 3D models to be classified into two categories: scaffold- and non-scaffold-based approaches [3]. The first requires an external support to be engineered to mimic the extracellular matrix (ECM), and this allows cells to anchor to the support, to proliferate, and migrate across scaffold interstices. Thanks to these characteristics, cells acquire typical in vivo tumor hallmarks, along with the specific distribution of an in vivo setting [4]. In non-scaffold-based platforms, tumor cells can aggregate and form so-called tumor spheroids. Therefore, the main aim of this method is to promote cancer-cell self-assembly by increasing interactions between the cells, adhesion to the support, and by amplifying cellular aggregation [5]. Finally, so-called “organ-on-a-chip” technology is an innovative 3D technology that is based on a multichannel microfluidic perfusion culture system [6] that can be made of different materials such as glass, plastic, and synthetic polymers. The system is composed of independent compartments in which different cell types are allowed to grow in the presence or absence of an ECM.

Since these tools are frequently used to evaluate the interconnections between cancer cells and the surrounding stroma, the enhancement of cell activity by integrating 3D cell cultures with functional, natural, and synthetic biomaterials is essential for the progressive maintenance of living-tissues architecture [7,8]. In particular, natural hydrogels better resemble native tissue-like properties compared to synthetic materials, and therefore are proposed as a more relevant support [9,10] to better recreate the biochemical and mechanical features of TME [11,12]. Due to the more realistic in vivo cancer environment of 3D cancer models compared to two-dimensional (2D) cancer models, we believe that drug-delivery systems (DDSs), principally stimuli-responsive DDSs, can significantly benefit from these in vitro preclinical models to evaluate the proper (temporal and spatial) delivery of a drug to its target site [13].

A DDS is described as a formulation or device via which a therapeutic substance is introduced into the body by the way of controlled delivery to improve drug efficacy and safety, and to monitor the rate, time, and place of drug delivery [14]. In this process, a therapeutic agent is administered and released from the delivery system at the target site, passing through biological membranes [15]. In other terms, a DDS represents an interface between a drug and a patient, and can ensure drug administration and tuned delivery for therapeutic purposes. Over the past 70 years, since the first DDS for dextroamphetamine sulfate (Dexedrine^®^) was produced by Smith, Kline, and French [16], three DDS generations have emerged: (i) basic DDSs for controlled release, including oral and transdermal sustained-release systems; (ii) smart DDSs, mainly developing nanotechnology-based DDSs; and (iii) modulated DDSs, which principally provide the targeted delivery of anticancer agents or siRNA to tumors, and long-term DDSs. All DDSs that belong to these generations have been designed with the goal of improving formulations for specific clinical purposes and for the treatment of various diseases [17]. Nanoparticles (NPs), which are designed to deliver the maximum possible amount of a drug to the target site, in particular to tumor tissues, have been the most popular DDS formulation over the last 20 years [18].

Several names have been used for NPs that are designed for drug delivery, and these include nanospheres, nanoconstructs, nanocarriers, and nanovesicles. These days, there is a consensus that the term “nanoparticle” represents all these formulations, including liposomes, polymer micelles, and emulsions [18]. NPs have been attracting so much interest in this field because they show “different” properties compared to micro/macro-particles, and these are commonly related to their huge surface area, and their ability to deliver both lipophilic and hydrophilic drugs to the target site. In this regard, two approaches have been used for successful drug delivery: passive and active targeting [19]. The first exploits the physiological conditions of the targeted organ or tissue to efficiently deliver the drug to the target site. For instance, in the case of NPs used in cancer therapy, passive targeting takes advantage of the exclusive pathophysiological architecture and features of tumor vessels, which are represented by leaky vasculature, with pores of 100–800 nm that enable NPs to accumulate in tumor tissues. Leaky vascularization, together with reduced lymphatic drainage, enhances NP permeation and retention within the tumor region thanks to the well-known enhanced permeability and retention (EPR) effect [20]. Therefore, drug-loaded NPs accumulate preferentially in tumor tissues rather than in normal tissues because NPs are not able to easily cross the blood capillaries of normal tissues, which are characterized by tight junctions, and this leads to a significantly higher drug amount in solid tumors than that of a free drug.

Active targeting refers to the attaching of ligands, such as vitamins, antibodies, aptamers, peptides, and small molecules, to the NP surface using a range of conjugation chemistry modalities, allowing them to exclusively bind, with high specificity, to a specific receptor on a cell’s surface. For this to be functional, receptor expression on tumor cells must be higher than that on normal cells [21]. Although all NP-DDSs against cancer have shown improved efficacy compared to the equivalent free (unloaded) drug in reducing tumor volumes and sizes in in vivo animal models, the translation of in vivo results to clinical practice has been very limited [18]. Indeed, animal models possess a superior physiological and biochemical relevance compared to in vitro models, but they are characterized by incomplete similarity to human phenotypic cancer heterogeneity [22]. In particular, mirroring the human TME complexity in pre-clinical models remains an ongoing challenge. We therefore feel that the translation of DDSs will be improved by using more appropriate preclinical models in their first stages of preclinical development, such as human 3D cancer models, especially for the development of endogenous and exogenous stimuli-responsive DDSs whose efficacy relies on the unique properties of TME.

**Figure 1:**
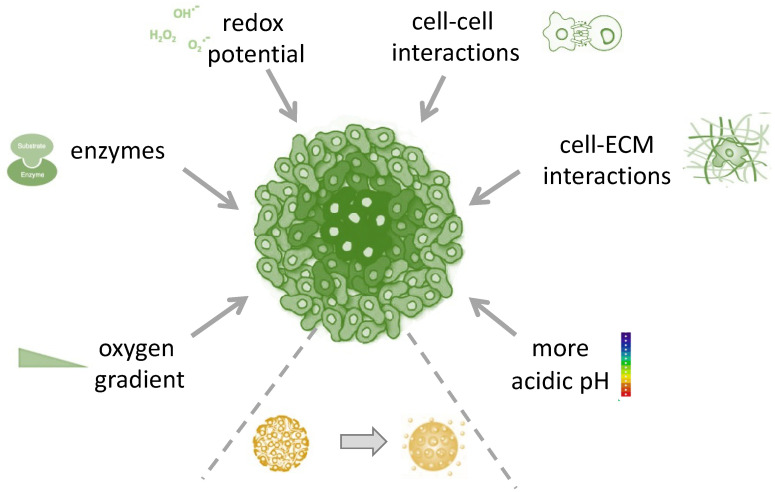
Endogenous triggers for site-specific delivery of drugs by stimuli-responsive drug-delivery systems.

**Figure 2:**
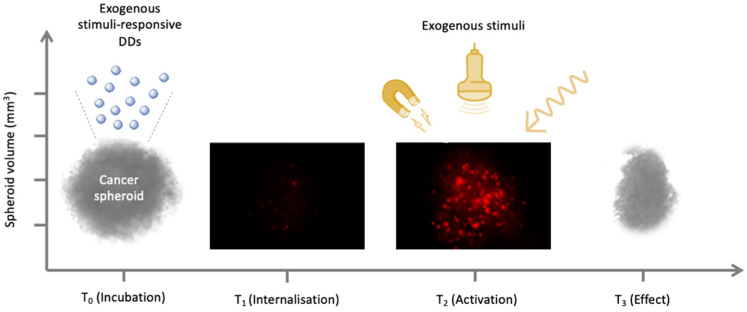
Exogenous triggers for time and site-specific delivery of drugs by stimuli-responsive drug-delivery systems.

The TME is a complex tissue that includes different cell types such as cancer-associated fibroblasts (CAF), cancer stem cells (CSCs), vascular endothelial cells, pericytes, and immune cells, along with non-cellular components such as the ECM and molecules secreted by the ECM. The existence of a strong symbiotic relationship between cancer and non-cancer cells has now been established, and it is known that this provides the permissive conditions for the growth and progression of malignant cells [23]. Moreover, it is well-known that the TME has unique physiological characteristics such as its pH, hypoxia, and the ability to up-regulate specific enzymes. Specifically, solid tumor extracellular pH (pHe) is more acidic (pH 6.5 to 6.8) than that of normal tissues. This is because cancer cells use glycolysis for energy consumption, rather than oxidative phosphorylation, in order to increase their biosynthetic function. This phenomenon, called the Warburg effect, leads to higher lactic acid production amount [24]. The low oxygen supply, called hypoxia, is caused by the atypical vascular network, which is not able to efficiently deliver blood to all the cells in TME [24]. Therefore, the improved comprehension of TME and its manipulation to make it less permissive towards tumor development are both currently of significant interest [25,26]. In this regard, DDSs have proven to be a promising platform for the disruption of the TME using cancer targeting either via endogenous stimuli, represented by the unique aberrant characteristics of the TME, such as pH, hypoxia, acidosis, redox potential, and enzyme alteration (Figure 1), or via exogenous stimuli, such as light, ultrasound (US), temperature, and ionizing radiation (Figure 2) [23,25,26,27,28]. Recently, stimuli-responsive DDSs have been developed by taking advantage of the unique properties of a novel class of fluorophores. Luminogens are non-emissive or weakly emissive agents in their molecularly dissolved state that emit intensively in their aggregated state owing to the restriction of the intramolecular motions (RIMs), and have been used as imaging-guided and pH-responsive DDSs for targeting therapy [29,30,31,32,33].

Since 2D cell-culture assays do not resemble the natural 3D cellular microenvironment, in vitro 3D models should be preferred when investigating stimuli-responsive DDSs. Moreover, 3D models can be even more realistic and controllable than human cancer animal xenografts where tumor cells are often transplanted to sites that, while convenient for tumor observation, do not reflect the microenvironment of the original tumor. Even when the transplantation site is orthotopic, differences between rodent and human physiology may result in a failure to establish the intricate TME. Despite these limitations, there are certain aspects of efficacy and toxicity that will always require evaluation in animal models prior to human clinical trials (Table 1). Furthermore, these facts, along with significant ethical concerns, have persuaded many research institutions to develop and take into consideration alternative models to reduce animal involvement and testing. In particular, cancer patient-derived organoids are 3D cultures able to recapitulate the inter- and intra-tumor heterogeneity, mirroring the cellular interactions of TME. These models have the potential to represent an innovative drug screening platform able to predict the clinical outcomes of new therapeutics [34,35].

Therefore, the aim of this review is to discuss the anticancer stimuli-responsive DDSs and their application in 3D cell-culture models (Table 2 and Table 3), and to show how the use of these in vitro 3D platforms is a promising, but still highly challenging method to close the gap between bench and bedside.

## 2. 3D In Vitro Platforms to Investigate Endogenous Stimuli-Responsive Drug-Delivery Systems

### 2.1. pH-Responsive Drug-Delivery Systems

It has long been recognized that inflammatory tissues and the TME are characterized by acidic pH (6.5). Indeed, the insufficient oxygen inside a tumor provokes hypoxia and leads to lactic acid production and ATP hydrolysis, causing an energy-deficient environment that contributes to an acidic hypoxia-driven TME [36]. However, cellular components, such as the endosomes, cytoplasm, endoplasmic reticulum, lysosomes, mitochondria, and Golgi bodies, are known to maintain their own pH values, from 4.5 in the lysosome to about 8.0 in the mitochondria. This means that, in tumor tissues, the pH is compartmentalized, with intracellular components being similar to normal tissues, and the extracellular component being somewhat acidic [37]. This difference in pH values between the intracellular and extracellular counterparts can therefore be exploited to deliver drugs that are weak electrolytes to tumors, using appropriate pKa values and a potential switch via pH-responsive DDSs [38]. For these DDSs, combined pH-sensitive linkages and polymers have been engineered to deliver a payload as the result of a pH-dependent conformational change via the destabilizing or decomposition of the DDSs in compartments with low pH [39].

The effects of the acidic hypoxia-driven TME have been extremely well evaluated in 2D cell cultures. However, although 2D cultures are easy to manage, they are mostly performed by incubating cells in specific gas-chambers or incubators where oxygen can be monitored progressively, meaning that these models fail to adequately recreate the TME with the appropriate oxygen and acidic gradients [40,41]. To overcome this drawback, researchers have sought tools for the design of 3D platforms to study the acidic hypoxia-driven TME, and several researchers have begun to use tumor spheroids to investigate their pH-responsive DDSs thanks to advances in this field [42,43]. In this regard, polymeric cluster nanoparticles (iCluster), which have been described by Li et al., are a perfect example of NPs being influenced by the differences in the pH present in tumor spheroids [44]. The higher extracellular acidity present in the 3D model triggered the discharge of poly (amidoamine) dendrimers (diameter ~5 nm), which were linked to a platinum prodrug. While NPs with a diameter of ~100 nm were retained at the periphery of the spheroid, the pH-mediated release of the dendrimers promoted their penetration into the spheroid, facilitating the increased cellular internalization of the therapeutic drugs [44]. A study by Swetha et al. has shown that histidine-modified star-shaped PLGA nanoparticles (sPLGA-His NPs), which contained docetaxel and disulfiram, exhibited more rapid drug release at a pH of 6.5 than that at a pH of 7.4 in colon cancer cells that were organized into both 2D and 3D cell culture models [45]. Moreover, the deep penetration of sPLGA-His NPs was observed in colon cancer spheroids, allowing authors to consider the use of this system as an effective tumor extra-cellular pH-responsive nanocarrier for efficient drug delivery to the tumor [45]. Interest has been aroused in nanogels, which are 3D hydrogels formed via the connection of nanoscopic micelles dispersed in an aqueous medium, for the treatment of brain tumors [46]. Nanogels are hydrophilic in nature, soft, biodegradable, and biocompatible, and one of their most important abilities is their pH sensitivity, meaning that they have been considered as pH-responsive DDSs. Their polymer networks/linkages are designed to undergo cleavage under low pH conditions, meaning that they completely degrade [47]. Yang et al. have developed a pH-triggered hyaluronic acid nanogel system by copolymerizing methacrylate hyaluronic acid with a cross linker that contains ortho ester groups [48]. The system itself carries doxorubicin, which shows excellent cancer cell uptake along with an enhanced anticancer activity in HepG2 human liver cancer cell spheroids. In particular, doxorubicin release was observed under endo/lysosomal conditions due to the pH-triggered cleavage of the ortho ester linkages [48].

### 2.2. Enzyme Responsive Drug Delivery Systems

It is now accepted that 2D and 3D cell-cultures are intrinsically different in their biological information; for example, an increase in enzyme activity has been observed in 3D cell cultures that more closely mimics the environment than 2D cell-cultures. Therefore, several works have evaluated enzyme-responsive DDSs evaluation in 3D models [49,50]. Indeed, these upregulated and/or altered enzyme expression profiles, which are common in cancer tissues, may potentially be exploited to control the release of cargo from nanocarriers and to monitor the cleavage of specific bonds between drugs and carriers in the TME [51,52]. In this regard, Kulkarni et al. have investigated the use of their pegylated nanovesicles as DDSs to release gemcitabine into 3D “tumor-like” spheroid cultures made from pancreatic ductal carcinoma cells (MIAPaCa-2 and PANC-1). This release is due to the destabilization of the pegylated nanovesicles under glutathione and metal-loproteinase-9 (MMP-9) action. The overexpression of the MMP-9 enzyme in the tumor ECM can chemically modulate drug delivery from the nanovesicles [53].

In another work by Liu et al., 4T1 breast cancer spheroids were developed to investigate the tumor penetration of large NPs, for imaging and photothermal purposes [54]. Large NPs have good retention ability, but are not able to reach zones that are distant from blood vessels, whereas small NPs can deeply penetrate tumor tissues, but are easily drawn back out into the blood stream [55]. For this reason, tumor-specific conditions, such as the overexpression of the enzyme hyaluronidase, are potentially useful as stimuli that can decrease NP size and allow them to more deeply penetrate tumors and perform drug release. In this regard, the authors built a large NP (AuNC@CBSA-ICG@HA) using hyaluronic acid (HA), which is a highly polymerized endogenous macromolecule that is degraded by hyaluronidase, in order to obtain tumor-specific hyaluronidase-sensitive size-reducible NPs [56,57]. Moreover, the HA shell also worked as a drug-release gate, with the tumor-specific hyaluronidase able to open the shell. Liu et al. showed that their large NPs were not completely able to homogenously distribute inside the spheroids, underlying that their initial large size influenced and prevented deep penetration. However, thanks to the degradation triggered by hyaluronidase, which shrunk the NPs, the penetration of NPs into 4T1 breast cancer spheroids was promoted. This data has also been supported by in vivo biodistribution experiments in a 4T1 mice breast cancer model, confirming the idea that the 3D model suitably reflects in vivo investigations [54].

In another work by Tan et a., a polymeric nanosized DDS (poly(OEGMA)-PTX@Ce6) that was made, for chemo-photodynamic therapy, of a cathepsin B-sensitive polymer-paclitaxel (PTX) prodrug and the photosensitizer chlorin e6 (Ce6) was engineered and investigated in 3D spheroids and, in in vivo, in BALB/C nude mice [58]. The main idea was to encapsulate the photosensitizer into NPs to facilitate cellular uptake, the generation of reactive oxygen species (ROS), and cancer cell killing under light irradiation with transitory low energy density. Since the incorporation of photosensitizers into nanomedicine makes them suitable for combinational therapies, the anti-tumor drug PTX was covalently linked to the polymer backbone via a cathepsin B-stimuli-responsive tetrapeptide Gly-Phe-Leu-Gly (GFLG), because cathepsin B, which is a lysosomal protease that is overexpressed in many cancer cells, is able to expedite the release of the covalently linked PTX [59]. The experiments were performed, as mentioned before, by creating 3D spheroids using human T24 bladder cancer cells. The authors observed good nanoplatform uptake into the spheroids followed by a significant induction in spheroid cell death, thanks to the synergistic activity between the light-activated photosensitizer and the chemotherapeutic drug PTX. Again, the authors also performed in vivo experiments to evaluate the therapeutic efficacy of their enzyme-responsive polymeric nano-DDS and, again, the in vivo results confirmed the observations obtained in the 3D spheroids, affirming that 3D models appear to be very useful for predicting in vivo results [58].

Finally, Hong et al. have used multi-cellular tumor spheroids (MCTS), made of human cervical adenocarcinoma cells (HeLa cells) and human alveolar adenocarcinoma cells (A549 cells), to investigate the dual enzyme/oxidation-responsive degradation of polyester-based NPs that were loaded with doxorubicin (Dox-NPs). The NPs had ester and sulphide linkages on their backbones, as esterase and ROS are present at higher concentrations in cancer cells than in healthy cells [60]. When the spheroids were incubated with Dox-NPs and then underwent esterase and hydrogen peroxide exposure, the NPs broke, allowing doxorubicin release and enhanced penetration into cells over time, up to four days after the incubation. Moreover, the fluorescence intensity uptake of the doxorubicin that was loaded into the NPs was five times higher than that of free incubated doxorubicin, which allowed the authors to confirm, thanks to the MCTS that developed, the ability of their nanoplatform to efficiently deliver and release the drug at a multicellular level [60].

### 2.3. Hypoxia-Responsive Drug Delivery System

Several regions inside solid tumors are characterized by low oxygen concentration (hypoxia) and areas of necrosis. The cells in these zones show resistance to chemotherapy and radiotherapy, and therefore also show aggressiveness and metastatic profiles that are characterized by poor therapeutic outcomes. However, these hypoxic regions also offer us the opportunity to investigate tumor-selective therapies, such as hypoxia-specific gene therapy, prodrugs that are activated by hypoxia, and hypoxia-responsive nanosystems [61]. In this way, 3D structures, mainly spheroids, are an ideal in vitro candidate with which to investigate and demonstrate the efficacy of hypoxic-sensitive DDSs. These 3D models show the hypoxic characteristics of solid tumors even when they are cultured under normoxia culture conditions, unlike 2D cell-cultures, as spheroids are characterized by high cellular density, with the inner cells being huge distances from the surrounding culture media, which limits nutrient uptake and drug diffusion through the 3D structures [62]. These features therefore make 3D models ideal candidates for investigating hypoxia-responsive DDSs, although many researchers prefer to investigate their hypoxia-responsive DDSs in cultured hypoxic spheroids [42,63]. In this regard, Kulkarni et al. have developed a DDS that forms polymersomes, made of polymer membranes, that are able to disintegrate under hypoxic conditions, allowing the release of encapsulated drugs in order to study the release of gemcitabine and erlotinib under hypoxic conditions [64]. The release of these two aforementioned anticancer drugs was investigated in a 3D cell culture model of BxPC-3 human pancreatic cancer cells, and the progressive release of gemcitabine and erlotinib, which were entrapped inside the polymersomes, was observed to be up to 90% under hypoxic conditions. Through this work, the authors were able to show that the concentration of oxygen in the near environment of the spheroids was able to influence their DDS, which showed a great potential of 3D cell culture models for future investigations into the treatment of hypoxic tumors [64].

In a work by Mamnoon et al., the authors studied their polymersomes, which were characterized by the incorporation of an estrogen receptor (ER) ligand onto the surface of the carrier, and the delivery of the anticancer drug doxorubicin into the hypoxic zones of ER-positive MCF-7 spheroids [65]. In particular, these spheroids were developed using a magnetic 3D cell-culture method for driving cell contact and spheroid formation. The data demonstrated that the targeted polymersomes have the potential to selectively target the ER-positive breast cancer cells, break into the hypoxic niches of the 3D spheroids, release nanoencapsulated doxorubicin, and reduce the MCF-7 breast cancer spheroid growth. Interestingly, although the classic MCF-7 monolayer cells showed the same cytotoxic trend as the MCF-7 breast cancer spheroids when treated with targeted polymersomes, there were differences when the non-targeted polymersomes were used. Indeed, the MCF-7 cells that were grown as 2D monolayers, both the targeted and non-targeted polymersomes showed statistically significant differences in cell viability when normoxic and hypoxic conditions where compared, whereas only targeted polymersomes showed a statistically significant difference in cell viability in MCF-7 breast cancer spheroids. This difference between the results of the MCF-7 monolayer and spheroids confirms that the monolayer culture, although offering extensive information, can sometimes mislead and/or overestimate results [65]. Finally, to further stress all of the benefits of working with 3D platforms in this field, we would like to point out that the cell viability data from the MCF-7 spheroids demonstrated that the targeted polymersomes were also able to shrink spheroid structures, relative to the untreated spheroids, under normoxic conditions [65]. This finding highlights how MCF-7 breast cancer spheroids are also able to intrinsically develop hypoxic conditions in normoxia culture conditions. This fact further closes the gap between in vitro and in vivo cancer models. 

### 2.4. ROS- and Redox-Responsive Drug Delivery Systems

The ability of ROS-responsive DDSs, in the form of nano- and microparticles, to ameliorate the therapeutic effects of drugs in the presence of huge amounts of ROS production is presently subject to intense investigation [66]. Briefly, these particles contain polymers that are functionalized with chemical groups that can react with ROS. When these particles are present in an oxidative setting, they progressively swell and eject their molecular cargo over time, or burst to discharge the entire amount of the drug all at once. The release of the molecular cargo from these particles can be influenced by abnormal ROS increases. Therefore, ROS-responsive particles can potentially minimize the side-effects that are caused by the off-target dispersal of therapeutic molecules into healthy tissues [67].

However, despite the interest in ROS-responsive DDSs, only one has been investigated in an in vitro 3D cell platform thus far [60,68]. This work has already been mentioned, in the section on enzyme-responsive DDSs, in this review. Indeed, Hong et al. have developed polyester-based NPs that are loaded with doxorubicin and bear sulphide and ester linkages on their backbones which, once exposed to esterase and ROS, are disrupted, allowing doxorubicin release. They investigated their DDS on MCTS from HeLa cells and A549 cells [60].

The same lack of investigations into stimuli-responsive DDSs in in vitro 3D models can be found in the redox-responsive DDS, in which the variation in the level of GSH in the tumor environment can be used for efficient internal stimuli-responsive drug release. To the best of our knowledge, no investigation into redox-responsive DDSs has yet been carried out on in vitro 3D models, although a work by Argenziano et al. has demonstrated the feasibility of using prostate cancer spheroids to investigate the biological effects of glutathione-responsive β-cyclodextrin-based nanosponges [69] (Table 2).

## 3. 3D In Vitro Platforms for Investigating Exogenous Stimuli-Responsive Drug Delivery Systems

### 3.1. Light-Responsive Drug Delivery Systems

Light radiation is an optimal non-invasive stimulus that allows the controlled and accurate treatment of a tumor to be performed. Photodynamic therapy (PDT) is an anticancer approach that exploits the synergic activity between a physical agent, in this case light, and a chemical agent, in this case a so-called photosensitizer (PS), which are some-times associated with a drug [70]. However, the success of PDT is limited by the poor penetration of the light into tumor tissues, the photosensitivity of the healthy cells that internalize the PS, and the difficulty inherent in administering PSs with low water solubility. In vitro 3D models and DDSs therefore provide considerable benefits for the development and testing of anticancer PDT regimens and, in fact, these new in vitro models have led to improvements in the investigation of PS accumulation in healthy tissues, which can avoid the significant adverse effects of photosensitivity [71,72]. We already mentioned a DDS that has been investigated using 3D spheroids for PTD purposes when we introduced enzyme-responsive DDSs. Indeed, Tan et al. have developed a polymeric nano-sized DDS that is characterized by a cathepsin B-sensitive polymer-PTX prodrug and a PS (chlorin e6, Ce6) for chemo-photodynamic therapy (poly(OEGMA)-PTX@Ce6) [58]. Their DDS is an enzyme-responsive polymeric nano-DDS that effectively carried PTX and the PS into tumor cells. The authors investigated the cellular uptake of the PS in the T24 bladder cancer spheroids using a photochemical internalization strategy, and the efficient delivery of the PS into the tumor cells was observed [58].

A combinational therapy that involves an anti-tumor drug and PDT has also been studied by Mozhi et al. This is a powerful and smart “all-in-one” nanoparticle-based DDS that can overcome biological barriers and leverage different cancer cell death mechanisms in a synergistic fashion. The engineered targeted micellar nanoprobe (TMNP) had a good encapsulation efficiency for simvastatin (SV), which is a hydrophobic drug, and protoporphyrin IX (PpIX), which was used as the PS [73]. The authors first carried out experiments on several cell lines, rat glioma C6, mouse brain-derived endothelial bEnd.3, mouse embryonic fibroblast NIH/3T3, and human umbilical vein HUVEC, which were cultured as 2D monolayers to investigate the cytotoxicity, underlying molecular mechanisms, blood-brain-barrier penetration, and the anti-angiogenic potential of TMNP. Moreover, although the results from the 2D mono-layer cell cultures were promising, Mozhi et al. also developed a 3D C6 multicellular tumor spheroid (MCS) as an intermediate step to more closely resemble in vivo studies and reduce the gap between 2D experiments and animal studies. The DDS developed by the authors showed good penetration and distribution and, under light exposure, a huge amount of ROS production, leading to cell damage via apoptotic and necrotic pathways in C6 MCS [73].

### 3.2. Temperature-Responsive Drug Delivery Systems

Temperature-responsive nanoplatforms are another example of exogenous-stimuli-responsive systems that have been exploited to create DDSs that sense and react to the surrounding environment. Thermo-responsiveness can lead to a sharp non-linear change in several properties of nanocarrier materials. The drug is released after a variation in the temperature of the surrounding microenvironment, allowing thermosensitive nanocarriers to maintain their load at body temperature (around 37 °C) and release it at higher temperatures (around 40–42 °C) to avoid rapid passage into the blood system and washout from the tumor [49]. Several researchers have investigated their temperature-responsive DDSs in 3D models, and these include Moreira et al. who introduced, as mentioned before in the pH-responsive DDSs section, a thermo- and pH-responsive carrier by incorporating doxorubicin into gold-core silica-shell nanorods that were associated with salicylic acid-loaded poly (lactic-co-glycolic acid)-based microparticles (NIMPS) [74]. Moreover, a gas-generating agent (NaHCO_3_) was also incorporated into the microparticles in order to provide them with pH-responsive activity. They tested the nanosystem on HeLa spheroids and it was shown that the combination of NIMPS and NIR laser irradiation for 10 min, with an increase in temperature of nearly 8 °C, resulted in more uniform doxorubicin distribution through the spheroids, which was observed in confocal laser scanning microscopy images, along with an increase in doxorubicin accumulation in the 3D structures. Moreover, the spheroid surface was slightly disorganized after the treatment and the margins started to disintegrate. Therefore, although the role of pH as a stimulus in drug release and response from their DDS was poorly investigated in the HeLa spheroids, the NIR laser irradiation was able to cause the drug release into the spheroids, indicating that improved efficacy may also be observed in in vivo animal models [74].

The response of temperature-sensitive DDSs has also been evaluated by Senavirathna et al. on human adenocarcinoma alveolar basal epithelial (A549) cells that were organized into a 3D model. The authors used low temperature-sensitive liposomes (LTSLs) that were loaded with doxorubicin in combination with mild hyperthermia (40–42 °C) and proton beam radiotherapy (PRBT) [75]. The combination of LTSLs and mild hyperthermia led to significant cytotoxicity when the spheroids were incubated at 42 °C. Specifically, LTSLs, under the heat trigger, were able to provoke a significant reduction in spheroid viability 72 h after the treatment, which is similar to the data obtained when spheroids underwent PBRT treatment. In this work, the authors observed how these A549 spheroids can be used as a platform for thermal and proton therapy [75].

### 3.3. Ultrasound-Responsive Drug Delivery Systems

The use of US as a triggering agent to release a drug at a specific target is appealing thanks to its non-invasiveness and the easily regulation of the physical parameters, such as frequency, the duty cycle and exposure time. The ability of US to facilitate drug release can be achieved several ways, but the most important is acoustic cavitation [76]. US consists of pressure waves, at a frequency of 20 kHz or greater, that can cause the release of a drug that is incorporated into a variety of DDSs, such as liposomal bubbles, microemulsions and metal NPs [77]. In fact, it has been demonstrated that the physical forces associated with cavitation can provoke the destabilization of nanocarriers along with drug release and a temporary increase in vessel permeability, resulting in the increased cellular uptake of therapeutic molecules [78,79].

Several articles have reported investigations into US-responsive DDSs in 3D models [80,81,82]. Logan et al. observed a significant reduction in MCF-7 spheroid volume, along with an increase in the number of necrotic cells when MCF-7 cells, which were organized into a 3D model, were treated with a combination of rose bengal, PTX, and doxorubicin, which were all loaded into a drug-delivery microbubble for the chemo-sonodynamic therapy of breast cancer [80]. This DDS was also able to induce a significant anticancer effect in mice bearing MCF-7 xenograft tumors under US exposure [80]. The fact that the same level of US-triggered DDS effectiveness was observed in both MCF-7 spheroids and an in vivo model strengthens the idea that 3D cell-culture models are suitable for use as in vitro models to predict the anticancer efficacy of stimuli-responsive DDSs [81]. This evidence was confirmed in another work by the same authors. Indeed, Logan et al. developed human pancreatic cancer (PANC-1) spheroids to study a gemcitabine-modified phospholipid, which was formed into a single PTX-loaded microbubble formulation, and the responsiveness to US. They performed these in vitro experiments before investigating the US-triggered DDSs on a human pancreas adenocarcinoma (BxPC-3) tumor that was ectopically implanted into a mouse model [82]. Their data once again confirmed the usefulness of 3D cancer spheroids in providing predictive results before investigations move to in vivo models, as the 3D model is characterized by a lower level of uncertainty than 2D cell-cultures, at least in regard to experimental setups in the field of sonodynamic therapy.

Finally, Grainger et al. have demonstrated that the penetration of NPs to the core of 3D breast cancer spheroids can be enhanced by pulsed US exposure in the presence of microbubbles, as it has been reported that microbubbles are able to reduce the cavitation threshold [83,84]. The authors also observed differences in NP concentration in the different spheroid layers in accordance with particle size, surface charge, and the US duty cycle used. Thanks to this study, the authors showed that the combination of pulsed US and microbubbles can be considered a synergistic tool that can allow nanocarriers to better penetrate solid tumors and thus facilitate their therapeutic effects [83].

### 3.4. Magnetic Field-Responsive Drug Delivery Systems

Magnetic NPs (MNPs) have shown promising results in several biomedical applications, and interest in 3D models for their investigation has risen in recent decades. The development of tumor-on-a-chip 3D systems relies on microfabrication, biomaterial research, microfluidics, and tissue engineering all together. These systems are characterized by microfluidic chips that allow cells to grow into 3D structures by controlling nutrients, waste removal, and small molecule supply. This technique can allow researchers to stimulate the essential features of NP transport, such as their uptake, dissemination into the extracellular matrix and extravasation into the tumor. Recent studies have illustrated how MNP behavior in response to magnetic fields can be deeply investigated using 3D microfluidic systems. For example, Benhala et al. have studied the movement of MNPs in response to an applied magnetic field on an on-chip system, and observed that all particles with a diameter size between 10 and 100 μm displayed the same trend; faster movement when larger in size, and slower movement when smaller [85]. Moreover, in another work by Geczy et al., it has been reported that MNPs are highly spherical in shape and exhibit superparamagnetic properties [86] (Table 3).

## 4. Conclusions

In this review, we have described the application of 3D models in the development of stimuli-responsive DDSs in order to understand whether this new in vitro approach might encourage the clinical translatability of these stimuli-responsive nanocarriers by improving their preclinical investigation. Several researchers have already tested their stimuli-responsive DDSs in the nanoscale and microscale range, mainly using cancer spheroids, to mimic the TME and nutrient supply, to estimate drug penetration and distribution, and, finally, to establish anticancer effectiveness more reliably.

It is very much worth noting that in works where 3D models were used before in vivo studies, the results of the in vitro and in vivo investigations were very similar. This confirms the suggestion that while 2D models offer valuable information, 3D models provide more accurate results that are predictive of the in vivo pharmacological features of DDSs. Nonetheless, despite the promising applications of cancer spheroids in these studies, the use of current 3D models is limited by several drawbacks, which include the cell type being able to influence spheroid formation, and limits to the model’s representation of a spheroid’s dynamic properties. Indeed, spheroid models do not account for transport across the vascular endothelium, thus limiting their ability to represent the EPR effect, which is a key feature in the success of DDSs in cancer treatment. Therefore, they rely exclusively on DDS diffusion to permeate cancer spheroids. Moreover, in the context of DDSs that are responsive to endogenous stimuli, the intrinsic features of the TME in cancer spheroids, such as pH, oxygenation, and enzymatic variations, are expressed at low levels, whereas they are lacking in 2D models, in which these features are doped. This consequently makes cancer spheroids less appealing to researchers than conventional monolayer cell cultures. Improvements in spheroid-development methods and investigations into stimuli-responsive DDSs in different types of 3D models, such as organoids and organ-on-chip systems, are therefore strongly encouraged, in particular as no experimentations on live cells that have been organized into a 3D structure has yet been performed with magnetic-responsive DDSs. These studies can boost the similarity between 3D models and real settings to improve the development of stimuli-responsive DDSs that are characterized by extreme selectivity of action both in space and time, with the aim of further closing the gap between bench and bedside in this promising scientific area.

## Figures and Tables

**Table 1:** Pros and cons of 2D and 3D in vitro and in vivo cancer models.

Model	Advantages	Disadvantages
2D in vitro models	Simple and low-cost maintenance Useful for drug screening and gene expression patterns	Partial polarization Few cell–cell and cell–matrix interactions Cells only adhere to one surface and migrate in one direction Do not reproduce cellular complexity and microenvironment network of tumor
3D in vitro models	Reproduce 3D architecture Increased cell–cell and cell–matrix interactions Different cellular migration in space Valuable for drug resistance mechanism	More expensive than in vitro 2D models More complex culture model
In vivo models	Higher reproducibility of physiological condition and well-known biology Easy development of tumor Study of cancer progression Reproducibility	Expensive Concerns regarding animal ethics Not all human targets have an animal homolog target

**Table 2:** Endogenous stimuli-responsive DDSs in 3D cancer models.

Endogenous	Stimuli	3D Model	Drug-Delivery Systems (DDSs)	Main Results	References
	pH	Polymeric cluster NPs (iCluster)	Polymeric cluster NPs (iCluster)	The pH-mediated release of the dendrimers promoted their penetration into the spheroid, facilitating the increased cellular drug internalization	[44]
		Histidine modified star-shaped PLGA (sPLGA-His NPs) loaded with docetaxel and disulfiram	Histidine modified star-shaped PLGA (sPLGA-His NPs) loaded with docetaxel and disulfiram	Docetaxel and disulfiram exhibited more rapid drug release by sPLGA-His NPs at pH 6.5 than at pH 7.4 in a 3D colon cancer model	[45]
		Human liver (HepG2) and human neuroblastoma (SH-SY5Y) spheroids	pH-triggered hyaluronic acid nanogel system by copolymerizing methacrylate hyaluronic acid with a cross linker that contains ortho ester groups that can deliver doxorubicin (DOX@HA-NGs)	Doxorubicin showed excellent cancer cell uptake when delivered as DOX@HA-NGs, along with enhanced anticancer activity	[48]
	Enzyme	Human pancreatic ductal (MIAPaCa-2 and PANC-1) carcinoma spheroids	Pegylated nanovesicles loaded with gemcitabine	Gemcitabine release is promoted by the destabilization of the pegylated nanovesicles under glutathione and metalloproteinase-9 action	[53]
		Human breast cancer (4T1) spheroids	Large NPs loaded with indocyanine green (ICG) and hyaluronic acid (HA) (AuNC@CBSA- ICG@HA)	Tumor overexpression of hyaluronidase allows a better penetration of NPs into 4T1 spheroids	[54]
		Human bladder (T24) spheroids	A cathepsin B-sensitive polymer-paclitaxel (PTX) prodrug and the photosensitizer chlorin e6 (Ce6) loaded into NPs (poly(OEGMA)-PTX@Ce6)	Ce6 released into T24 spheroids and then light irradiated produced ROS. The PTX was also released by cathepsin B, determining anticancer effect	[58]
		Human cervical adenocarcinoma (HeLa cells) and human alveolar adenocarcinoma (A549 cells) spheroids	Polyester-based nanoparticles loaded with doxorubicin (Dox-NPs)	Spheroids incubated with Dox-NPs and then exposed to esterase and hydrogen peroxide, underwent to increased Dox penetration and fluorescence intensity	[60]
	Hypoxia	Human pancreatic (BxPC-3) spheroids	Polymersomes loaded with gemcitabine and erlotinib	Gemcitabine and erlotinib, entrapped into polymersomes, were released under spheroid hypoxic condition up to 90%	[64]
		Human breast cancer (MCF-7) spheroids	Polymersomes with an estrogen receptor (ER) ligand incorporated onto the surface of the carrier to deliver doxorubicin (E2-Dox-HRPS)	Targeted polymersomes showed a difference in cell viability in normoxic and hypoxic condition	[65]
	ROS	Human cervical adenocarcinoma (HeLa cells) and human alveolar adenocarcinoma (A549 cells) spheroids	Polyester-based nanoparticles loaded with doxorubicin (Dox-NPs)	Spheroids incubated with Dox-NPs and then exposed to esterase and hydrogen peroxide incubation, underwent to increased Dox penetration and fluorescence intensity	[60]

**Table 3:** Exogenous stimuli-responsive DDSs in 3D cancer models.

Exogenous	Stimuli	3D Model	Drug Delivery Systems (DDSs)	Main Results	References
	Light	Human bladder (T24) spheroids	A cathepsin B-sensitive polymer-paclitaxel (PTX) prodrug and the photosensitizer chlorin e6 (Ce6) loaded into NPs (poly(OEGMA)-PTX@Ce6)	Ce6 released into the T24 spheroids and then light irradiated produced ROS. The PTX was also released by cathepsin B, determining anticancer effect	[58]
		Rat C6 glioma cell line (C6 MCS)	Targeted Micellar Nanoprobe (TMNP) with exceptionally high encapsulation efficiencies of a hydrophobic drug simvastatin (SV) and a photosensitizer protoporphyrin IX (PpIX)	TMNP under light irradiation showed huge amount of ROS production and induction in apoptotic and necrotic pathways	[73]
	Temperature	Human cervical (HeLa) spheroids	Doxorubicin-loaded gold-core silica-shell nanorods with salicylic acid-loaded poly (lactic-co-glycolic acid)-based microparticles (NIMPS)	Uniform doxorubicin distribution under NIR irradiation along with spheroid surface disorganization	[74]
		Adenocarcinomic human alveolar basal epithelial (A549) spheroids	Low temperature-sensitive liposomes (LTSLs) loaded with doxorubicin	Combination between LTSLs and mild hyperthermia induced reduction in spheroid viability	[75]
	Ultrasound	Human breast cancer spheroids (MCF-7)	Microbubbles loaded with rose bengal, paclitaxel, and doxorubicin (O_2_MB-PTX-Dox/O_2_MB-PTX-RB)	MCF-7 spheroid volume reduction and increase in necrotic cells under sonodynamic exposure of O_2_MB-PTX-Dox/O_2_MB-PTX-RB	[80]
		Human pancreatic (BxPC-3) spheroids	Gemcitabine-modified phospholipid incorporated into a single microbubble formulation loaded with PTX (Lipid-Gem-PTX MB)	A statistically significant reduction in BxPC-3 spheroid volume was observed when spheroids underwent to US exposure of Lipid-Gem-PTX MB	[82]
	Magnetic	On-chip system	Magnetic NPs (MNPs)	Particles with a diameter size between 10 and 100 μm displayed the similar trend	[85]
		Microfluidic chip	Magnetic NPs (MNPs)	MNPs showed highly spherical shape and superparamagnetic properties in the system	[86]
